# A multicentre randomized controlled trial of food supplement intervention for wasting children in Indonesia-study protocol

**DOI:** 10.1186/s12889-019-6608-5

**Published:** 2019-03-13

**Authors:** Aria Kekalih, Indriani Oka Anak Agung Sagung, Umi Fahmida, Evi Ermayani, Muchtaruddin Mansyur

**Affiliations:** 1 Community Medicine Department, Faculty of Medicine, Universitas Indonesia, Jl. Pegangsaan Timur No.16, RT.1/RW.1, Pegangsaan, Menteng, Kota Jakarta Pusat, Daerah Khusus Ibukota, Jakarta, 10310 Indonesia; 2 Southeast Asian Ministers of Education Regional Centre for Food and Nutrition (SEAMEO RECFON), Pusat Kajian Gizi Regional Universitas Indonesia, Jl. Salemba Raya 6, Jakarta Pusat, 10430 Indonesia

**Keywords:** Food supplementation, Programme evaluation, Infant and young child feeding, Randomized controlled trial

## Abstract

**Background:**

After the first six months of exclusive breastfeeding, children are introduced to liquids and semi-solid food, known as the complementary feeding phase. This phase is critical because it is often accompanied by improper feeding in children, which may lead to wasting and other nutrition problems. Fortified biscuits have been provided for wasting children as a nationwide programme. However, the ability of children to accept food supplementation remains questionable. This paper describes the protocol of a study investigating the efficacy of food supplementation (PMT biscuit) and nutrition education to improve the nutritional status of wasting children in Indonesia.

**Method:**

The efficacy of a government food supplementation programme will be examined using a randomized control trial design. Parents with wasting children aged 6–17 months will be recruited to participate in the study. After obtaining informed consent and pre-intervention measures, participants will be assigned into three arms of intervention with PMT biscuits and/or nutrition education only. The two primary outcomes for this study are the nutritional status of wasting children and PMT biscuit compliance. Characteristics of all subjects in each arm will be analysed and compared with each other to assess their comparability at the beginning. The data will be collected at pre-intervention, at 3 months of intervention, post-intervention, and at the 6- to 9-month follow up.

**Discussion:**

This paper aims to describe the study protocol of a randomized controlled trial investigating the effects of different PMT biscuit portion and nutrition education in two arms and nutrition education only in another arm. This study is important because it will provide evidence for the Indonesian government regarding the efficacy of food supplementation and/or food-based recommendations to improve the nutritional status of wasting children aged 6–23 months in Indonesia.

**Trial registration:**

The study has been registered at clinicaltrials.gov, maintained by the National Library of Medicine (NLM) at the National Institutes of Health (NIH), on April 26, 2018, and was last updated on April 30, 2018 (registration number: NCT03509155).

## Background

Children under 5 years of age undergo a period of rapid growth and development. Therefore, this age group needs attention because of their vulnerability to malnutrition. In Indonesia, children under 5 years of age with undernutrition (weight-per-age) and wasting (weight-per-height) remain greater than 10%. Feeding practices in compliance with the WHO standard is a concern that needs to be addressed using a community health programme approach [1, 2].

According to the concept of the first 1000 days of life, the possibility of improvement opportunities for a child that develops undernutrition or wasting problems can only be exploited within the first 2 years. Thus, the chances of the child achieving optimal growth within the next age range will be greater [3]. Food supplementation is comprehensively provided by the Indonesian government to pregnant mothers, children under 5 years of age, and school children to improve their nutritional status to achieve their best physical and intelligent development.

In the first program, food supplementation activity was provided in the form of a cash transfer to identified vulnerable families to enable them to have a suitable food supplement for their families. In 2001, the cash transfer programme was then modified to food supplementation of factory-made biscuits using a special formulation that was fortified with vitamins and minerals. This food supplementation was designed for children aged 6–59 months whose nutritional status fell into the wasting category (weight-for-height less than − 2 SD). The food supplementation was suggested to be given alongside breast milk and their daily food. The biscuit provided to children under 5 years of age was designed to add nutritional needs required to reach the age-appropriate weight. Food supplementation for children under 5 years of age was given to the children who were not indicated for hospitalization.

After the first 6 months of exclusive breastfeeding, children are introduced to liquid and semi-solid food. Children aged above 1 year are introduced to solid food or a family diet [4]. With most undernutrition children coming from low-socioeconomic families, a concern was raised whether this food supplementation will create another form of dependency as in the Indian study of the Ready to Use Therapeutic Food (RUTF) programme [5]. Thus, the sustainability of families and mothers to provide good nutritious food for children during and after the time of food supplementation needs to be assessed.

Another aspect to be explored is assessing whether the efficacy of food supplementation to improve the nutritional status of wasting children in multiple cities to describe Indonesian geographical and socio-economic diversity is necessary. This randomized controlled trial is aimed to assess the efficacy of food supplementation provided by the government and/or food-based recommendations in improving the nutritional status of wasting children aged 6–23 months in Indonesia.

### Characteristics of the fortified biscuits as a food supplement

**Fig. 1:**
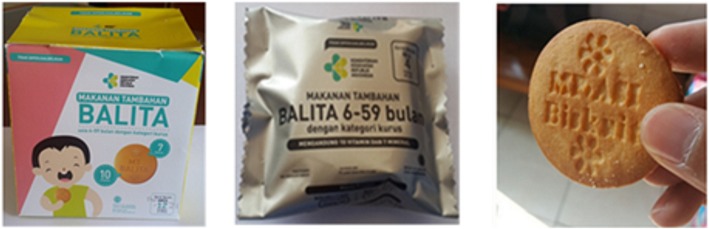
Packaging content and form

The characteristics of the fortified biscuits are based on their texture, taste, colour, weight, safety, and expiration date. The biscuits have a crunchy texture that softens immediately when mixed with a liquid (water or milk), and they have a sweet taste and a normal cook-processed (not burnt) colour. The biscuits are prepared to meet the quality and safety requirements for infants and young children. All the products expire 24 months after production. The product should be eligible for consumption at the time between the production date and expiration date. The biscuit box uses three-layered packaging to ease the storage and provision of separate portions, as shown in Fig. 1.

The primary package contains four biscuits with weights no less than 40 g containing 160 cal, 3.2–4.8 g of protein, and 4–7.2 g of fat. The food supplementation biscuits are enriched with ten vitamins (A, D, E, K, B1, B2, B3, B6, B12, and folic acid) and seven minerals (iron, iodine, zinc, calcium, sodium, selenium, and phosphorus). One secondary box contains twenty-one primary packages weighing 840 g. One tertiary packaging contains 4 secondary packages.

Technical instructions for biscuit provisions as food supplementation are clearly mentioned in the tertiary package and include the targeted children, dose, and serving. Supplemental food in the form of biscuits is indicated for children aged 6–59 months who are wasting (weight-for-height less than − 2 SD). Children aged 6–11 months and 12–59 months are suggested to consume eight biscuits (2 packages) per day and 12 biscuits (3 packages) per day, respectively. The children underwent monthly weight gain monitoring in the village-based integrated growth monitoring post *(pos pelayanan terpadu = posyandu).*

Food supplementation is stopped if the child has reached the appropriate weight and is suggested to continue with balanced nutrition family food. Biscuits could be either directly consumed or in the form of mushy food by adding more water onto the biscuits in a clean bowl. To maintain their well-nourished condition, children are recommended to have balanced nutrition appropriate for their age.

## Methods/design

This study is an open-label randomized controlled trial with three parallel arms for the intervention of food supplementation (PMT biscuit) management. The design of this study was developed using SPIRIT guidelines by Chan A-W et al. [6]. The first arm was performed as a control group obtaining only routine IYCF counselling by the health cadres and without the provision of biscuits, while the second arm was similar to the first intervention group but received fortified biscuits with a portion as recommended by the Ministry of Health and was given IYCF counselling by the cadres and nutritionist. The third arm was similar to the second intervention group but received biscuits with an adjustment in portions and IYCF counselling that emphasized the optimization of local food. IYCF counselling promoted complementary feeding recommendation (CFR) that was optimized using a linear programming approach. Adjustment in the biscuit portions in the third arm is calculated based on the nutrient gap between the nutrient requirements and optimized CFR.

In the 2nd and 3rd arms, the intervention is administered for 3 months according to the recommended duration of biscuit delivery by the Ministry of Health. Monitoring is conducted every month during treatment and will be followed in the sixth and ninth months from the beginning of the intervention.

### Study subjects

This study uses the following inclusion criteria: children aged 6–17 months with a weight-for-length z score less than 2.00 to − 2.99 considering that the children are still categorized as children under 2 years of age at the end of the intervention. The other criterion is that they do not receive the biscuits at the time of recruitment and the subjects’ parents agree to participate in the research by signing the informed consent document.

During the determination of study eligibility, four populations were identified that are inappropriate for entry into the food supplementation (biscuit) randomized controlled trial, including the severe wasting (weight-for-length less than − 3 Z-score) population because it needs to be treated differently and because it is strongly associated with a very high short-term mortality risk [7].

The exclusion criterion of this study is a severe food insecurity status of the households because their access to nutritional food is severely hampered, affecting not only the young children but also all family members.

Despite poor dietary intake, infection is also considered a determinant of nutritional status; therefore, having tuberculosis infection (based on anamnesis) is also used to exclude the subjects. Having TB infection as a co-infection in parallel with acute malnutrition was found to be a predictor of child mortality [8].

To minimize the loss to follow-up subjects, the possibility of moving to other cities within 6 months of intervention is also listed as an exclusion criterion. All the terms and conditions of the study will be explained before the caregiver/parents sign the inform consent documents.

Adverse events will be recorded and immediately reported to the PIC through the centres’ coordinators, and those affected will be referred to the nearest public health centres involved in the study. All severe adverse events will be reported to the Health Research Ethics Committee of FKUI/RSCM.

### Data to be collected

**Fig. 2:**
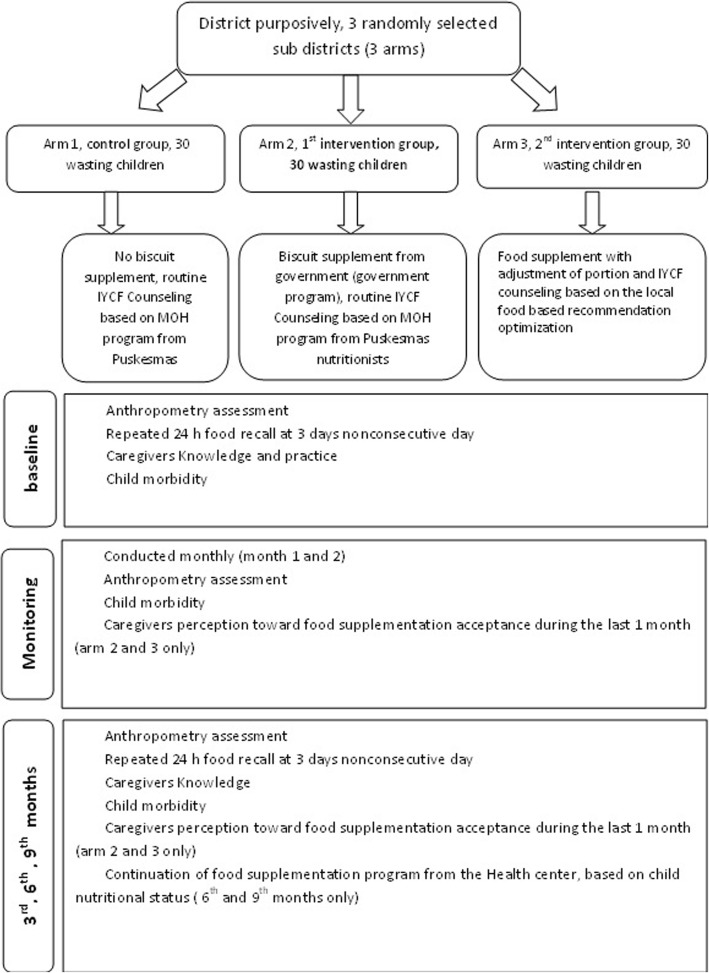
Flowchart of the data collection

The following data are collected based on the study subjects: subject characteristics (age, sex, and history of disease) and anthropometry (body weight and body height) data. Other data collected from the subjects’ mother/caregiver include the subject characteristics (demographic, socioeconomic, house condition), food intake of the child [using 24-h food recall for 3 days and last 1 week food frequency questionnaire (FFQ)], and perceptions of the supplemental biscuits (packaging and delivery method). The same method is also used to collect data for compliance with feeding instructions, exposure to information (health information sources, health information ever obtained in Posyandu/health services post), and IYCF-related knowledge. The flowchart for data collection is shown in Fig. 2.

### Sample size

The sample size is calculated using the two means comparison formula. Based on Afarwuah, the standard deviation is obtained at 0.95 [9]. The sample size calculation is performed using the 95% confidence level, 80% power, and an estimated difference in the weight-for-length Z-score and drop-out of 0.30 and 20%, respectively. Based on the formula and data used above for the minimal sample size, 540 subjects are required for this study. Considering the feasibility of subject and study implementation, 90 subjects per district are required: 30 subjects as a control group or arm 1, and 30 subjects each as intervention group 1 or 2 (arm 2 or 3). Thus, a minimum of 6 districts is needed to meet the minimum sample size required.

### Sampling method

**Fig. 3:**
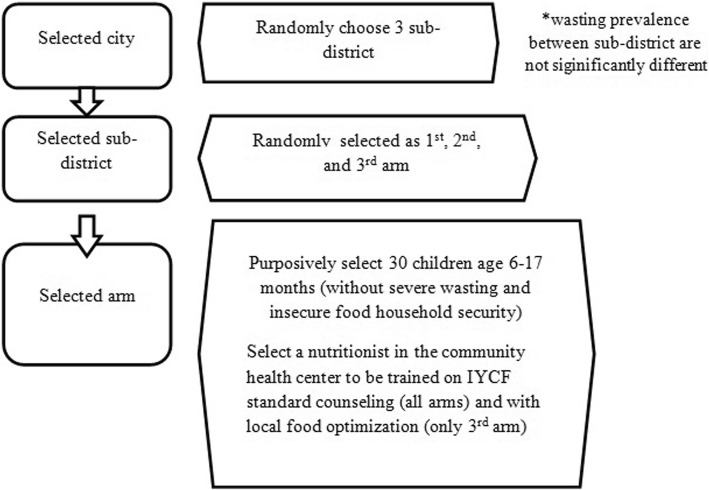
Sampling method

The sampling procedure is started by selecting provinces and districts in cooperation with the Ministry of Health. Next, among sub-districts with a moderate prevalence of wasting children, three are selected to become arms of the study. The prevalence rate of moderate wasting is 10–15%, and the decision to include moderate wasting is to maintain the heterogeneity conditions of the selected sub-districts. Three selected sub-districts are then selected randomly to be assigned as arm 1 (control) and arms 2 and 3 (intervention 1 and 2, respectively). Subjects are generated from *posyandu*, from which only those with 2–5 eligible children were chosen. Thus, to fulfil the total number of 30 subjects, each sub-district involves 6–15 *posyandu*. Expansion to another sub-district will be done when there are fewer than 30 wasting children aged 6–17 months in each selected sub-district. The sampling method scheme is provided in Fig. 3.

This study comprises three arms, which are explained as follows:

- Arm 1 No Intervention: Control group.

The first arm is performed as a control group and does not receive biscuits but undergoes the routine IYCF counselling programme by a nutritionist from the health centre.

- Arm 2 First intervention group. This arm receives biscuits with portions as recommended by the Ministry of Health and obtains IYCF counselling by the nutritionist.

Subjects aged 6–11 months and 12–13 months should consume eight and twelve supplementation biscuits per day, respectively, based on the recommendation from the Ministry of Health, accompanied by participation in the routine IYCF counselling programme by the nutritionist from the health centre.

- Arm 3 Second intervention group (experimental). This arm receives the adjusted number of biscuits based on nutritional gap analysis.

The subject should consume four biscuits per day, for both children aged 6–11 months and 12–23 months. IYCF counselling is delivered with a specific message based on the nutrition gap analysis.

Due to the nature of the intervention, neither participants nor the health staff can be blinded to allocation but must not disclose the allocation status of the participant at the follow-up assessments.

### Patient and public involvement

This study would not involve any patient because participants who need medical treatment and have a severe food insecurity status of the households will be excluded. The results of the study will not be entirely disseminated to the study participants; nevertheless, the results of the anthropometric assessment and its changes will be provided to the participants’ mothers as a part of nutrition education. The health cadres will be trained at the preparation stage and involved in the study as an accompanying *Puskesmas* nutrition educator.

### Outcome measures

- Primary: Nutritional status of wasting children.

The measurement will be recorded through study completion, from baseline and at third, sixth, and ninth months. Anthropometry measurements will be based on the weight-for-length indicator (WHO Child Growth Standards).

- Secondary: PMT biscuit compliance.

The measurement will be recorded through study completion, at the first to third months after the start of treatment and then at the sixth and ninth months from the beginning of treatment.

### Portion size adjustment according to nutritional gap analysis

The nutrient gap is a gap between the nutrient requirement and optimized nutrient intake that is identified using linear programming (LP) analysis and Optifood software using data regarding children under 2 years of age from a nationally representative study in Indonesia. LP analysis will identify problem nutrient(s), nutrient-dense food groups, subgroups, and food, as well as the optimized complementary feeding recommendation (CFR) [4]. The percentage of RDA gaps is calculated as the nutrient requirement subtracted by the percentage of nutrient requirement from the best diet in the LP analysis. The gap is converted into the required number of biscuits to ensure all nutrients can be fulfilled by the intake of complementary food plus the biscuits [10].

LP analysis found that the portion size of the biscuit provision should be reduced to only four biscuits for both children aged 6–11 months and aged 12–23 months. However, this new adjustment should be accompanied by IYCF practices with an emphasis on local food optimization. IYCF counselling is provided monthly by a nutritionist at Primary Health Care (PHC/*Puskesmas*) accompanied by cadres.

### Counselling of optimized CFR

The counselling session is conducted once a month for the first 3 months. Each education session takes 30–60 min prior to the distribution of food supplementation. Training to involve *Puskesmas*’s nutritionists and health cadres in the study area is conducted at the preparation stage to comprehend the nutrition counselling material for each arm. Every participant receives printed nutrition-promoting materials related to appropriate messages for each arm. The monthly meeting is also gathered to update field conditions during counselling.

The optimized CFR material and IYCF final version are sent by SEAMEO RECFON to each centre to be printed. The IYCF materials are prepared by the Ministry of Health, WHO and MCAI [11] with modifications of counselling materials initially targeting for 0–24 months of age.

### Assessing food insecurity in households

The assessment of food insecurity in households is conducted as a preliminary assessment to screen the subjects using structured interviews of the United States Household Food Security Survey Module (US-HFSSM) questionnaire, which focuses on assessing the ability of households to meet basic food needs and the subject’s habits and responses to the condition. There are 18 tiered questions to be given to the subject. These questions are also used to ensure that the poor nutritional status involved does not come from the severe food-insecure household. The categories of household food insecurity are based on the score of 0–2 as food secure, 3–7 as low food security, and 8–18 as very low food security.

### Anthropometry assessment

This study collects data on the weight and height of children to identify the nutritional status and characteristics of the children. This assessment was also used at the screening stage to select children according to the criteria. Wasting is defined as weight for a Z-score length less than − 2.00 [12].

Anthropometric training is conducted prior to data collection to ensure adequate precision and measurement accuracy—i.e., technical error of measurement (TEM) ≤0.7 cm and bias ≤0.7 cm compared with the results of criterion anthropometric measurements [13]. One researcher from each centre receives anthropometric training to standardize the measurement. Next, at each centre, the researcher trains all the data collectors and research assistants to perform the weight and length measurements (the calibration and placement of appropriate tools, the correct position of the subjects for measurement, correct reading, instructions to the subjects being measured, and recording the measurement results) [14].

#### Measurement of body weight

Body weight is measured in kilograms using SECA weight scales. The study maintains tools and measurements to ensure internal validity using the following considerations: the storage of tools must be kept in the horizontal position and shall always be put at the top of the pack, or at least, the items on it shall not exceed one kg.

Calibration of the instrument is performed daily to ensure that the tools work properly. The calibration uses a 1-kg calibration weight or 2 one-litre water mineral bottles as a substitute for the calibration weight. The weight of two mineral water bottles should not exceed a 0.01 kg difference between each measurement. The weighing is performed two times and always recorded in a special calibration note.

During the measurement process, the device should be used on a flat surface to maintain the stability of the measurement. Prior to weight measurement, the child should remove accessories that can affect the weight as well as height, such as shoes, socks, t-shirts, slings, hair pouches, hats or hoods. Diapers should also be removed before weighing and can be re-applied after weighing is complete. The research assistant explains to the mother/caregiver the process of weighing in advance.

Body weight is measured to the nearest 0.1 kg. The weight measurement is performed twice by a trained anthropometry team. When the difference between the first and second measurement is more than 0.2 kg, a 3rd measurement is performed. Two closest values are taken as the measurement result. Guidelines to determine the nutritional status of children are used from a decree that was released by the Ministry of Health of Indonesia in 2010 [14].

#### Measurement of body length

Height in children under 2 years is known as body length because the measurement is performed in the supine position. The instrument used to measure the length of the child is the Shortboard, with a precision of 0.1 cm. The length measurement requires at least two assistants and the mother/nanny. One person keeps the head touching the headboard and eyes at the Frankfurt plane position, the mother/caregiver in the middle is asked to hold the chest and lower thighs to keep the body straight, and one person is at the base, holding the mobile board to perform the measurement.

A flat area, slightly spacious and with sufficient lighting are required for length measurement. Like weight measurement, clothing or accessories that may affect the measurement results should be removed such as socks, shoes, hair pouches, hats or hoods. This process should be performed quickly because the child usually feels uncomfortable and wriggling makes it difficult to measure. The measurement is performed twice and so is the recording.

### Dietary assessment

Measurement of intake is performed using the (1) one-week-FFQ (Food Frequency Questionnaire), to measure the intake of nutrient-dense foods identified in optimized CFR, and (2) 24-h recall for 3 non-consecutive days (2 weekdays, 1 weekend), to measure the usual intake of macro and micronutrients in the population and measure the proportion of subjects at risk for inadequate iron intake and other micronutrients. The 24-h dietary recall interview is performed using a multiple-pass 24-h dietary recall [15]. The data are gathered from caregivers/mothers by mentioning all types of food consumed on the previous day. To obtain the same perception of food size, a standard “Food Photos Book” is used.

### Assessment of the perception of packaging and mode of delivery of supplemental biscuits

This question assesses the mother’s perception towards the supplemental biscuits to their children for the last 1 month. Assessment is performed using a Likert scale (best score = 5; 1 = least good score). The questions posed are the taste of PMT biscuits, suitability of the biscuit packaging in the form of biscuits given to the child according to the age of mother, suitability of the biscuit packaging (wrap and colour) regarding whether it is interesting and explanation to the mother how to feed the biscuits.

### Compliance to feeding instructions

Compliance assessment is performed by filling the record form given to the caregiver by the enumerators. Caregivers receive a monthly record form and are asked to complete the record every day. They should write down a reason when the biscuit eaten is less than the recommendation (only for 2nd and 3rd arm). The compliance form is collected monthly by the field team. The categories of compliance are grouped as follows: (1) Good, > 90%, (2) Medium, 80–90%; (3) Low, 60–80% and (4) Noncompliant, < 60%.

### Assessment of knowledge and practice based on IYCF

The infant and child feeding practices of caregivers are measured based on IYCF key indicators recommended by WHO/UNICEF and include dietary diversity, minimum meal frequency, and minimum acceptable diet, while the assessment of knowledge is generated from the practice indicators. There are ten questions of knowledge in the form of true-false questions.

### Place and time of execution

Data collection will be conducted in 7 selected sites in Indonesia in accordance with Ministry of Health Coordination (Sambas Regency - West Borneo [1.3625° N, 109.2832° E], Depok Municipality – West Java [6°24′8.94″S, 106°47′39.27″E], Padang Pariaman Regency – West Sumatra[0.5547° S, 100.2152° E], Sleman Regency – Yogyakarta [7.7325° S, 110.4024° E], Banjar Baru Municipality – South Borneo [3°27′26.07″S, 114°48′37.14″E], Sidoarjo Regency – East Java [7.4726° S, 112.6675° E], Makassar Municipality – South Sulawesi [5°8′39.76″S, 119°25′35.9″E]). Data from each site are collected by investigators from 7 research centres of respected universities in Indonesia (SEAMEO RECFON, Faculty of Public Health Universitas Indonesia; Faculty of Public Health Andalas University; Faculty of Public Health Lambung Mangkurat University; Faculty of Medicine, Public Health, and Nursing Universitas Gadjah Mada; Faculty of Public Health Airlangga University; Faculty of Public Health Hasanuddin University). SEAMEO RECFON serves as a lead investigator. This study is planned for 3 months using a grant scheme in 2017 so that implementation in the 6th and 9th months will be continued in 2018. The enrolment of the study participants was initiated in January 2018 and finalized in April 2018. It is expected that the final data collection would be in December 2018.

### Plan of data analysis and presentation

All data analysis will be performed using SPSS version 20. The data of the study subjects, both children characteristics and mother/caregiver characteristics, will be analysed to obtain the distribution frequencies and central tendencies as appropriate to the data scale. All subject characteristics of each arm will be analysed and compared with each other to assess their comparability at the beginning. No missing data are expected, and all loss to follow-up study participants will be excluded. The comparison of the weight-per-length score between groups and between times will be performed using ANOVA. The study subjects’ compliance to take biscuits will also be analysed and reported. The final dataset will be accessible to all authors without masking.

The research team is responsible to make annual and final reports to the Ministry of Health. The dissemination of the study results will be presented at the national nutritional meetings in the three regions. The scientific publications are the rights and responsibility of the research team.

### Data monitoring committee

A committee to monitor data collection has been established and comprises an advisory board of experts at SEAMEO RECFON. The data monitoring committee (DMC) is not involved in the study as a team member. The DMC advises the study team members regarding the continuing safety of trial subjects as well as the continuing validity and scientific merit of the trial.

## Discussion

Food supplementation in the form of a biscuit has been established for a decade as a national programme to reduce wasting prevalence in Indonesia. The official daily serving size of the biscuit is considered very dense in energy and another macronutrients. Unfortunately, information on its effectiveness is lacking, and the concern of the energy dense nature provided might inhibit another nutrient. The decision of food supplementation in the form of biscuits and the serving needed to be consumed daily have not been supported experimental study-based evidence in the community. There is a concern that if the amount of biscuit given is too energy dense, it may inhibit the absorption of other nutrients. This study intends to examine the effectiveness of consuming supplementary biscuits and provide alternative strategies that can achieve optimal results. Additionally, an alternative strategy is proposed in this study by modifying the daily serving size and highlighting the use of local based food in a nutrition education session.

## Abbreviations

CFR: Complementary feeding recommendation
DMC: Data monitoring committee
FFQ: Food frequency questionnaire
IYCF: Infant and young child feeding
LP: Linear programming
MCIA: Millennium challenge account – indonesia
PHC: Primary Health Care
PMT: Food supplementation (*Pemberian Makanan Tambahan*)
RUTF: Ready to Use Therapeutic Food
TB: Tuberculosis
TEM: Technical error of measurement
US-HFSSM: United State Household Food Security Survey Module
WHO: World Health Organization
